# Cytokines and chemokines: The vital role they play in herpes simplex virus mucosal immunology

**DOI:** 10.3389/fimmu.2022.936235

**Published:** 2022-09-23

**Authors:** Jacinta B. Smith, Jason J. Herbert, Naomi R. Truong, Anthony L. Cunningham

**Affiliations:** ^1^ Centre for Virus Research, The Westmead Institute for Medical Research, Sydney, NSW, Australia; ^2^ Faculty of Medicine and Health, The University of Sydney, Sydney, NSW, Australia

**Keywords:** HSV, chemokines, cytokines, mucosal, immunology

## Abstract

Herpes simplex viruses (HSV) types 1 and 2 are ubiquitous infections in humans. They cause orofacial and genital herpes with occasional severe complications. HSV2 also predisposes individuals to infection with HIV. There is currently no vaccine or immunotherapy for these diseases. Understanding the immunopathogenesis of HSV infections is essential to progress towards these goals. Both HSV viruses result in initial infections in two major sites - in the skin or mucosa, either after initial infection or recurrence, and in the dorsal root or trigeminal ganglia where the viruses establish latency. HSV1 can also cause recurrent infection in the eye. At all of these sites immune cells respond to control infection. T cells and resident dendritic cells (DCs) in the skin/mucosa and around reactivating neurones in the ganglia, as well as keratinocytes in the skin and mucosa, are major sources of cytokines and chemokines. Cytokines such as the Type I and II interferons synergise in their local antiviral effects. Chemokines such as CCL2, 3 and 4 are found in lesion vesicle fluid, but their exact role in determining the interactions between epidermal and dermal DCs and with resident memory and infiltrating CD4 and CD8 T cells in the skin/mucosa is unclear. Even less is known about these mechanisms in the ganglia. Here we review the data on known sources and actions of these cytokines and chemokines at cellular and tissue level and indicate their potential for preventative and therapeutic interventions.

## Introduction

Herpes simplex viruses type 1 and 2 (HSV1 and 2) are members of the *Alphaherpesvirinae* family and are known for their broad tissue tropism and ability to establish and maintain latency in sensory ganglia (1–3). HSV1 and 2 typically initiate primary infection by gaining access to the deep layers of the epidermis of oropharyngeal and genital mucosal tissue. From these infection sites HSV spreads into the axons of sensory neurons in the epidermis and then travels towards the trigeminal/dorsal root ganglia where it establishes a lifelong latent infection, protected from immune eradication. After recurrence HSV1 & 2 usually cause self-limiting lesions which are primarily found around the mouth, face and eye (HSV1) or genital area (HSV1 and 2), or asymptomatic infection. Occasional severe complications include encephalitis, blindness, predisposition to HIV acquisition, and in severe cases in neonates and immunosuppressed individuals, death. HSV1 and 2 are both highly prevalent in the human population with (as of 2016) 3.7 billion people globally under the age of 50 infected with HSV1 and 491 million people infected with HSV2 (4). Most HSV1 and 2 spread to others occurs through contact with an asymptomatic actively shedding individual through saliva or genital secretions.

**Figure 1:**
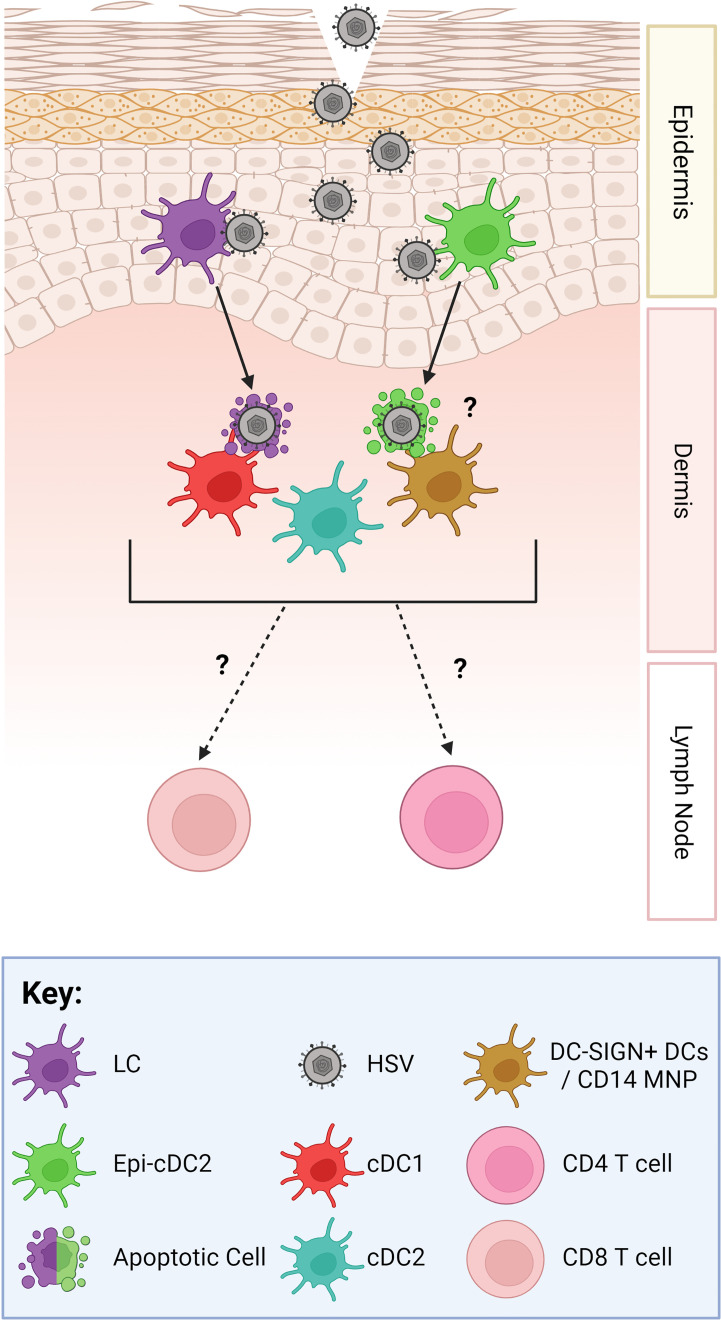
The Human HSV Viral Relay. As HSV gains access to the epidermis, hypothesised through micro-abrasions, it infects LCs (purple) and Epi-cDC2s (green). LCs then migrate down into the dermis, undergo apoptosis, and cluster with and are taken up by dermal DCs, such as cDC1s (red) and DC-SIGN ^+^ DCs/macrophages which are termed CD14 MNPs (mononuclear phagocytes) (orange) (9). Dermal cDC2s (blue) have not yet been shown to cluster with LCs. Epi-cDC2s have been shown to undergo apoptosis but whether they cluster with the dermal DCs is yet to be shown. Subsets of murine dermal DCs carry HSV antigen out of the dermis and have been shown to present the HSV antigen to CD4 and CD8 T cells in the lymph node (10–12). However, this has not yet been shown in human studies.

HSV1 and 2 reactivate asymptomatically very frequently with occasional noticeable lesions in both oral and genital locations. Reactivation is continually suppressed by the immune system to ensure that reactivation periods are limited. Several types of immune cells and their products function to contain and clear HSV infections such as keratinocytes, which produce chemokines that recruit T cells to the site of infection (5), infiltrating CD4 T cells which secrete interferon-γ (IFN-γ) (6) and effector CD8 T cells that clear infected lesions *via* direct killing or IFN-γ control of HSV-infected cells (7). These immune responses are initiated by the initial infection of Langerhans cells (LCs) and probably by epidermal type 2 conventional dendritic cells (Epi-cDC2s) in the epidermis (8). LCs undergo apoptosis and migrate to the dermis where they transfer HSV antigen to non-infected dendritic cells (DC) in the dermis in a virus “relay” (9), summarised in **Figure 1**. All these processes require the secretion and actions of cytokines and chemokines. Cytokines and chemokines are key soluble modulators of immune cells and are secreted initially by resident epidermal and dermal cells – LCs, DCs, macrophages, and keratinocytes; then later by infiltrating immune cells. Some of these communications can be hijacked and modulated by HSV to allow it to further its infection and counteract antiviral immune responses, which will be discussed below. However unlike other Herpesviruses, HSV has not been shown to produce any functional cytokine and chemokine homologs (13).

Cytokines are crucial modulators and communicators between immune cells and non-immune cells, and as such they play a vital role in all functions of the immune system. Cytokines are best known for regulating local and systemic inflammation; however, they can also be involved in cellular migration, cellular proliferation, and wound repair processes (14, 15). As cytokines are mostly involved in regulating inflammation, they are crucial in host defence against disease, and as such there are both pro- and anti- inflammatory cytokines. Cytokines and cytokine receptors can be categorised into several superfamilies depending on their biological activity and molecular structure. Cytokines include interleukins, (denoted by an “IL”), and the superfamilies; interferons (IFN: e.g. IFN-α, β, and γ), tumour necrosis factors (TNF: TNF-α and Lymphotoxin), growth stimulating factors (including granulocyte macrophage-colony stimulating-factor (GM-CSF), and chemokines (16). These superfamilies consist of structurally related but not always functionally similar cytokines.

Chemokines orchestrate cellular migration in the immune system and are an integral part of the immune response. Chemokines are secreted by all the major immune cells into the extracellular space, and remain as either soluble proteins or bind to extracellular matrix components, forming a chemotactic gradient to attract other cells (17). Chemokines are divided into four different families, depending on the location of the first four conserved cysteines in the ligand: CXC, CC, C and CX3C (18–21). Each family of chemokines has corresponding G protein-coupled receptors and ligands (22). Chemokines can also be classified as either inflammatory or homeostatic in their nature (22, 23).

The aim of this review is to examine and collate published data on the cytokines and chemokines produced by immune cells in response to HSV infection and their role in the immune response against HSV. Additionally, the cytokine and chemokine responses to HSV in infected mucosal tissues and the role cytokines and chemokines play in HSV vaccine design will be reviewed. We have specifically compared findings in *in vivo* murine models, human *ex vivo* explants and occasionally biopsies of human herpes lesions to help define relevance to human disease and vaccine development.

## Identification of cytokines and chemokines in human recurrent herpes lesions

Primary HSV lesions are an understudied resource as they are rarely obtained. Primary herpetic lesion biopsies can provide unparalleled information of the initial immune response HSV stimulates in human tissue, that cannot be discovered in animal or cell culture models. The role of cytokines and chemokines are yet to be investigated in human primary lesions. The cytokines and chemokines present in recurrent lesions have been studied. However due to the ever-expanding knowledge of immunology and the development of higher parameter technologies, not all cytokines and chemokines were investigated in past studies. Studies of human recurrent lesions showed IL-1α, IL-1β, IL-6, IL-10, IL-12, CCL4, CCL3 and CCL5 were detected on Day 1 post onset. IL-1β and IL-10 levels were highest out of the cytokines, while CCL4 levels were significantly higher than the other chemokines. By Day 3, TNF was detected while levels of IL-1β, IL-6 and CCL4 were decreased compared to Day 1. Levels of IL-10, IL-12 and CCL3 were comparable to those seen on Day 1 (5). Studies of recurrent HSV lesions are still lacking key data, including a comprehensive study of cytokines and chemokines and data for timepoints longer than 3 days. Such work still needs to be conducted, and as such is a major gap in the literature.

## Immune cells and the role their cytokines and chemokines play in HSV infection

**Figure 2:**
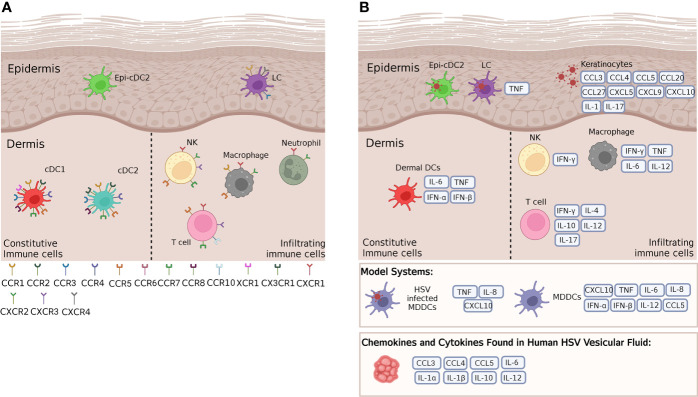
Cytokines and chemokines and their receptors expressed by key cutaneous and mucosal immune cells. **(A)** A wide range of chemokine receptors are expressed on cutaneous immune cells in steady state, in both murine and human mucosal tissues. These receptors respond to a variety of differing chemokines, some of which are crucial in HSV infection and immunity. **(B)** According to mouse and human cell culture models, HSV infected murine LCs produce TNF, while HSV infected MDDCs produce a variety of cytokines and chemokines such as TNF and CXCL10, and uninfected MDDCs alone have been shown to produce a variety of cytokines and chemokines. These models provide a guide to the cytokines and chemokines that human LCs, Epi-cDC2s and dermal DCs may produce in response to HSV infection in the skin. In the epidermis, infected keratinocytes produce many cytokines and chemokines that attract infiltrating cells to the site of infection. In the dermis, murine dermal DCs have been shown to produce IL-6, TNF and Type I IFNs, while resident and infiltrating immune cells such as NK cells, T cells and macrophages produce a plethora of cytokines and chemokines in response to HSV infection of other immune cells. Many of these same cytokines and chemokines have also been found in human HSV vesicular fluid. For further details of which cytokines and chemokines are produced by human and/or murine models please refer to **Table 1**.

**Figure 3:**
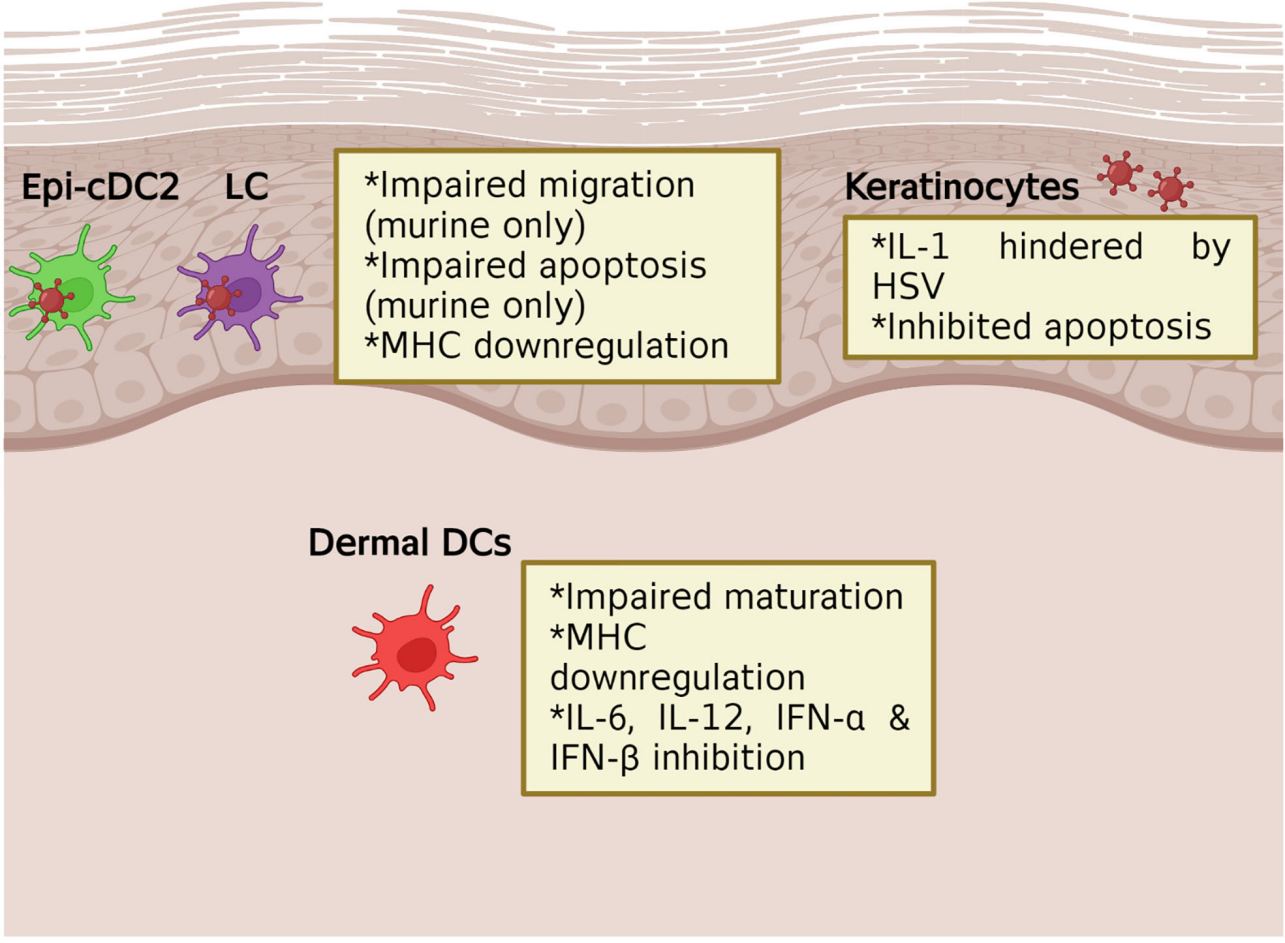
The impact of HSV on initial immune cells production of cytokines and chemokines. In murine models, HSV infection of the initial immune cells, such as LCs, has been shown to lead to impaired migration and apoptosis. This leads to downstream effects seen in dermal DCs where migration and cytokine and chemokine production is inhibited. HSV infection of keratinocytes has also been shown to inhibit apoptosis and the production of IL-1.

**Figure 4:**
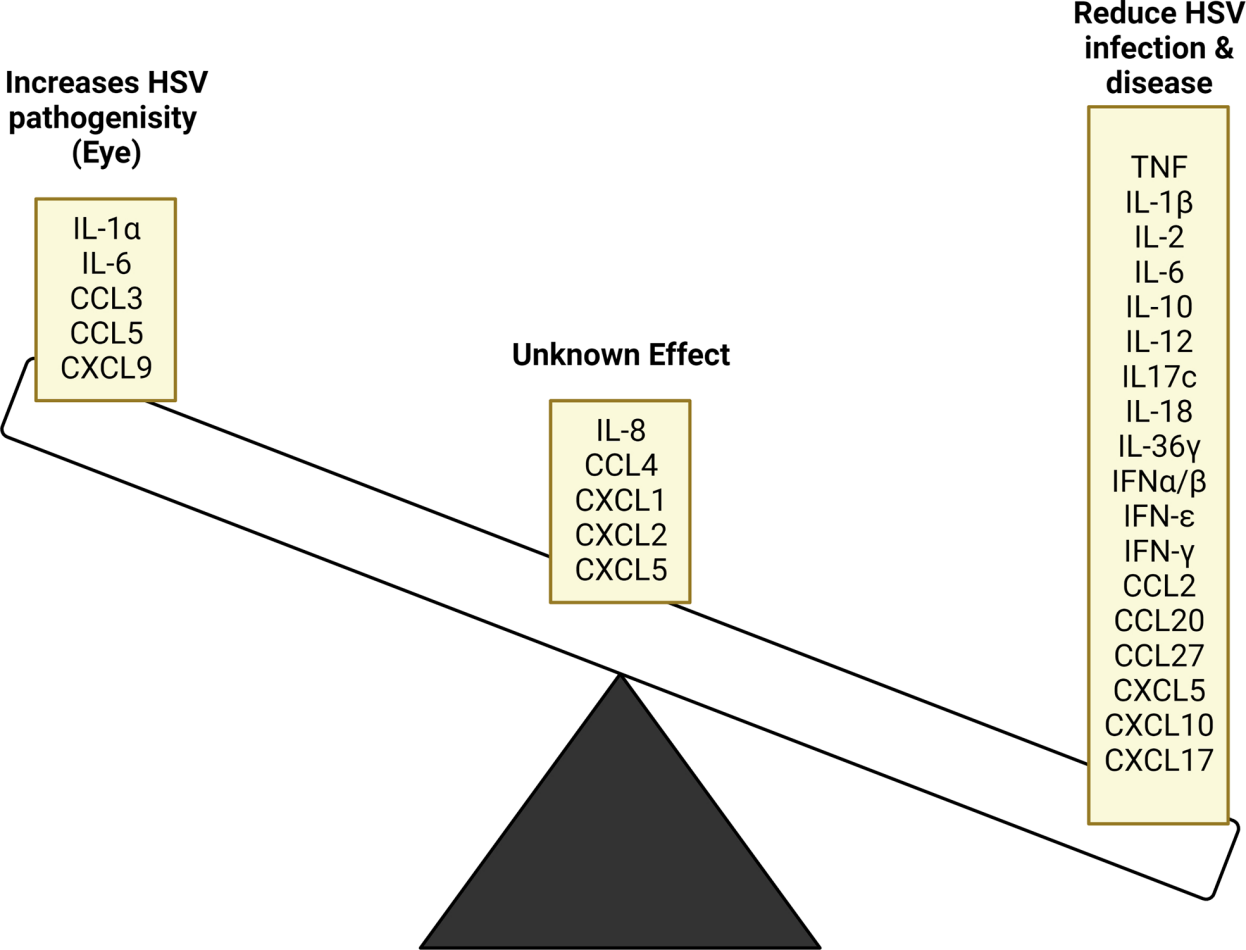
Cytokine and chemokine inhibitory and stimulatory effects on HSV replication and disease. HSV infection stimulates the production of various cytokine and chemokines, many of which act to supress viral replication. In some instances, these products have contradictory outcomes based on the tissue site being affected, while others require further study to determine their role. In ocular infection, the stimulated cytokines and chemokines released further HSV pathogenicity instead of resolving infection.

Oropharyngeal, genital skin and mucosal tissues are divided into the superficial epidermis and the deeper dermis. Various resident immune cells are found in these tissues, LCs and a new type of conventional dendritic cell, Epi-cDC2s are found in the epidermis, while a wider variety of resident immune cells (e.g. DCs, CD4 and CD8 T cells, macrophages, natural killer (NK) cells, gamma-delta T cells) are found in the dermis (24). All of these cells produce cytokines and chemokines in relation to HSV infection, which will be examined in this section. **Figure 2** summarises the main cytokine and chemokine responses from each immune cell and **Figure 3** highlights the effects HSV has on initial immune cells and the chemokines and cytokines they produce. **Figure 4** summarises each effect of these cytokines and chemokines on HSV replication and disease, while **Table 1** highlights the cytokines and chemokines produced by HSV infection and their roles in the immune response.

**Table 1:** Cytokines and chemokines produced during murine or human HSV infection and their subsequent role in immunopathogenesis.

Cell Type	Cytokine/Chemokine	Effect in HSV infection	Murine or Human	References
Keratinocytes	TNF	Reduces viral spread in surrounding keratinocytes, increases release of complement factors, increases CXCL5 and CCL20 expression	Human	(25–27)
	IL-1	Reduces viral spread in surrounding keratinocytes, increases CXCL5 and CCL20 expression	Human	(25, 27, 28)
	IL-6	Reduces viral spread in surrounding keratinocytes	Human	(25, 28)
	Type I IFN (IFN- α/β)	Reduces viral spread in surrounding keratinocytes, increases MHC-I expression on keratinocytes, increases apoptosis in keratinocytes	Human	(29–33)
	CCL3	Role unknown	Human	(5, 34–36)
	CCL4	Role unknown	Human	(5, 34–36)
	CCL5	Recruitment of T cells, retention of CD4 T_RM_s in vaginal tissues	Human	(5, 34–36,)
	CXCL9	Recruitment of CD8 T cells towards the epidermis	Human	(5, 34–37)
	CXCL10	Recruitment of CD8 T cells towards the epidermis	Human	(5, 34–37)
	CXCL5	Recruitment of neutrophils	Human	(5, 34–36, 38)
	CCL20	Recruitment of Th17 T cells	Human	(5, 34–36)
	CCL27	Likely increases T cell retention in tissue post infection	Human	(5, 34–36, 39)
Dendritic Cells (MDDC/DCs)				
HSV infected MDDCs	TNF	Increases CCR5 expression on surrounding cells, which may enhance migration of DCs but also increases HIV susceptibility	Human	(35)
	CXCL10	Recruitment of CD8 T cells	Human	(40)
	IL-8	Role unknown	Human	(41)
Bystander DCs/MDDCs	CCL5	Recruitment of CD4 T cells, retention of CD4 T_RM_ cells in vaginal tissues	Human	(40, 42)
	CXCL10	Recruitment of CD8 T cells	Human	(40)
	IL-6	Role unknown	Murine	(43)
	IL-8	Role unknown	Human	(43)
	IL-12	Initiates differentiation of CD4 T cells towards a Th1 subtype	Murine & Human	(43)
	Type I IFNs (IFN-α & IFN-β)	Role unknown	Human	(40)
	TNF	Role unknown	Human	(40)
Plasmacytoid Dendritic Cells (pDCs)	Type I IFN (IFN- α/β)	Antiviral state	Human	(44)
	IL-18	Amplifies IFN-γ secretion by NK cells	Murine	(45)
	TNF	Recruitment and activation of NK cells and T cells	Human	(46)
	IL-6	Recruitment and activation of NK cells and T cells	Human	(46)
	CXCL10	Recruitment and activation of NK cells and T cells	Human	(46)
	CCL3	Recruitment and activation of NK cells and T cells	Human	(46)
Macrophages	TNF	Role unknown	Murine	(47, 48)
	IFN- α	Role unknown	Murine	(47, 48)
	IL-6	Role unknown	Murine	(47, 48)
	IL-12	Induces IFN-γ in NK cells	Murine	(49)
NK Cells	IFN-γ	Antiviral effector that enhances MHC-I and -II expression on keratinocytes, increases TLR3 expression on keratinocytes, activates macrophages and DCs, stimulates B cells	Human	(48)
T cells				
CD4 T cells	IFN-γ	Limits HSV replication, activates B cells, stimulates keratinocytes to produce CXCL9 and CXCL10	Human & Murine	(50–54)
CD4 Th1 cells	IL-2	Regulates neutrophil invasion in the cornea and prohibits CD4 Th17 cells	Murine	(55, 56)
CD8 T cells	IFN-γ	Promotes DCs presenting antigen to CD8 T cells	Human	(57)
Tregs	IL-10	Suppression of CD4 and CD8 T cell proliferation, suppression of IL-2, IL-6 and CCL3 production	Murine	(58, 59)

### Keratinocytes

While not usually considered an immune cell, keratinocytes can detect pathogens, including HSV through pattern recognition receptors (PRRs) such as Toll-like receptors (TLRs). Components of HSV can bind to and be detected by TLR2, TLR3 and TLR9, resulting in activation of early innate immune antiviral responses (25, 60). Keratinocytes are the first cells to be infected by HSV, this is initially detected by TLR2 binding of viral glycoproteins (26). TLR2 signalling induction results in the production of pro-inflammatory cytokines including TNF, IL-1, IL-6, and antiviral cytokines such as Type I interferons (25, 28). These cytokines can act on the surrounding keratinocytes and immune cells to reduce viral spread. IFN-α/β induces an antiviral state in surrounding keratinocytes and increases MHC-I expression on the HSV infected keratinocytes allowing them to be more easily targeted and eliminated by CD8 T cells (29, 30). IFN-α/β also stimulates apoptosis. However HSV inhibits apoptosis and opposes this effect in most cell types (31–33). IL-1 is produced by keratinocytes, but its release is hindered by HSV infection. IL-1β, a proinflammatory cytokine, is active in its cleaved form but HSV suppresses this activation. IL-1ɑ is biologically active in its pro form with its release further encouraging inflammation and neutrophil infiltration (61). Another indirect effect of TLR2 stimulation is the enhancement of tight junctions between keratinocytes to reduce virus spread through intercellular interstices (62). Keratinocytes secrete complement factors, C3a and C5a constitutively at low levels, and TNF increases this expression, resulting in chemokine release which recruits innate immune cells (63). Infiltrating T and NK cells produce IFN-γ which also increases TLR3 expression on keratinocytes (64). As HSV infection progresses, keratinocytes have been observed to express HLA-DR after stimulation with IFN-γ released from CD4 T cells. This results in further interaction and subsequent activation of T cells, reducing viral spread (30). Indeed, synergy between the IFN-α, -β and -γ produced from differing cell types may result in synergistic antiviral action which controls HSV spread, particularly after exit from axon termini as seen in recurrent HSV models (65, 66). Keratinocytes also express IL17c during HSV infection, which can protect neurons from infection and is suggested to promote Th17 activity (67, 68). However, in HSV induced stromal keratitis of the eye, Th17 cells were observed to enhance the immunopathologic effect (56).

Keratinocytes produce several chemokines in response to HSV including CCL3, CCL4, CCL5, CXCL9, CXCL10, CXCL5, CCL20 and CCL27 (5, 34–36). CXCL9 and CXCL10 facilitate the recruitment of T cells towards the epidermis. Uninfected keratinocytes produce these mediators at basal levels, but the response to infectious HSV particles and early production of IFN-γ by innate immune cells amplifies their expression (37). In mice a loss of CXCL9 or CXCL10 has been observed to increase viral load and suggests redundancies between CXCR3 ligands (69). TNF and IL-1 together increase the production of CXCL5 and CCL20 (27). CXCL5 acts on CXCR2 which is highly expressed by neutrophils (38). Recruitment of neutrophils to HSV infection sites is well recorded but their benefit is questionable as some studies indicate that neutrophils may enhance damage and worsen disease severity (70). CCL27 production by keratinocytes has been observed, although its expression has not yet been examined in lesions (5, 36, 39). CCL27 acts to recruit T cells to inflamed tissue and has redundancies with CCR4 ligands (34).

Overall, the keratinocyte response to HSV is complex and crucial in early infection. They produce many cytokines upon interaction with HSV, combined with increased production of chemokines released after interaction with other resident and infiltrating immune cells. Cross-signalling between them is critical for optimisation of early host defences and HSV control.

### Dendritic cells

DCs are a crucial part of the immune response to HSV because of their ability to bridge the gap between the innate and adaptive immune system. Their main role in infections is to patrol for pathogens, which upon detection, they engulf and present antigen to naive T cells in lymph nodes.

LCs are one of the first immune cells to encounter HSV, thus making them vital for the HSV immune response (10). As discussed previously, LCs are infected by HSV and migrate down into the dermis where they cluster with dermal DCs, the cells that then present HSV antigens to T cells (9, 71). It is hypothesised that cytokines and chemokines are involved in this process, but little is known about the chemokines and cytokines that could be involved. HSV infected human monocyte derived DCs (MDDCs) have been shown to produce TNF, CXCL10 and IL-8, factors that could be important in the clustering of LCs and DCs. Interestingly, TNF has been shown to upregulate CCR5 on surrounding DCs (and LCs) making them more susceptible to coinfection with HIV (41) and may enhance migration of DCs towards CCR5 ligands produced by HSV infected keratinocytes.

Previously LCs were the only identified immune cell in non-inflamed epidermis, but our recent studies have shown that another immune cell resides in the epidermis, Epi-cDC2s. Epi-cD2s are the predominant cell type in anogenital tissues (e.g. vagina and foreskin). The exact role of Epi-cDC2s in the HSV viral relay is yet to be elucidated, however we have shown them to be highly infected by HSV in tissue explants and *in vitro* (8). Little is known about which cytokines and chemokines they produce in response to HSV; however, they were shown to produce several cytokines when stimulated with a cocktail of TLR agonists, some of which are key cytokines in the HSV immune response including IL-1β, IL-6 & IL-10 (72).

Human tissue is hard to access, and processing is a complex procedure, meaning most work on HSV infection has been conducted in MDDCs. Cytokine and chemokine responses of skin and mucosal DCs may differ from MDDCs. To-date there has been no comprehensive characterization of the cytokines and chemokines produced and detected by skin DCs in steady state and HSV infection. For MDDCs it has been shown they can express a variety of cytokine and chemokine receptors (i.e., CCR1, CCR2, CCR3, CCR4, CCR5, CCR7, CCR8, XCR1, CX3CR1) (73–77). MDDCs have also been shown to produce a wide variety of cytokines and chemokines including CCL2, CCL3, CCL4, CCL5, CXCL9, CXCL10, CCL17, CCL18, CCL22, under a range of conditions and stimulations (74) (See **Table 1** for detailed description of function). At least some of these cytokines and chemokines will be produced or utilised by skin/mucosal DCs in HSV infection as well, specifically in the migration of infected LCs. Although model human MDDCs infected with HSV *in vitro* show impaired migration in response to CCL19 and to upregulate CCR7 (78–80), this is not complete with authentic epidermal LCs, particularly in humans. In mice a proportion of infected LCs remain trapped in the epidermis but others migrate into the dermis, carrying HSV (10). However in human skin explant models (and biopsied recurrent HSV lesions) HSV infection induces the migration of apoptosing LCs to the dermis where they interact with clusters of dermal DCs and are taken up by them (9). Whether these LCs and also Epi-cDC2s migrate towards particular chemokines expressed during infection or the dermal DCs are attracted to infected LCs *via* chemokines needs to be investigated.

Several studies conducted in murine models have also been able to elucidate cytokines and chemokines that DCs release in relation to HSV infection. However, some of the data has not been investigated *in vivo* meaning there might be differences between these seen *in vitro*. A study by Sato et al. using bone marrow derived DCs showed secretion of IL-6 and IL-12 in response to infection with specific HSV1 KOS_TLR2*_ variants, through either TLR2 or TLR9 stimulation (81, 82). Similar to this, human MDDCs treated with HSV2 dUTPases have also been shown to produce a wide variety of proinflammatory cytokines including IL-6, IL-8 and IL-12, specifically through TLR2 activation (43). Regarding TLR9 another study showed that CD11c^+^ cDC2s in TLR9 knockout mice produced similar levels of IFN-α, but reduced levels of TNF and IL-6 compared to WT mice (83). These studies show how stimulating various TLRs results in a wide variety of cytokines produced.

Human MDDCs have also been shown to produce cytokines inducing Type I and Type III IFNs (IFN-α, IFN-β and IL-28 or IFN-Λ), TNF and chemokines (CCL5 and CXCL10) in response to HSV1 infection (40). HSV2 infected MDDCs have also been shown to produce TNF and IL-6, Type I and III IFNs, some IL-10 but reduced expression of IL-1β and IL-12 (84). The differences in chemokine and cytokine production in human MDDCs in the reported studies could be due to differences in virus strains used or in experimental designs. Murine CD11c^+^ cDC2s have also been shown to express Type I and III IFNs, as transgenic CD11c-DTR-tg mice, vaginally infected with HSV2, showed decreased levels of Type I and III IFNs when their CD11c^+^ DCs were depleted (85).

As well as releasing their own chemokines and cytokines, DCs also stimulate T cells to release cytokines and chemokines. One study in the vaginal tract of HSV2 infected mice showed that inflammatory monocyte derived DCs were required to help Th1 CD4 T cells secrete IFN-γ (86). This process was reliant on Type I IFN signalling and CCR2 mediated migration of the inflammatory monocytes to the vaginal tract (86). Another study showed that vaginal submucosal CD11c^+^ DCs were important in the stimulation of CD4 T cells to release IFN-γ, when mice were infected with HSV2 (87). Thus, regardless of the origin and type of DC, they are crucial for stimulation of an effective antiviral T cell response.

HSV has a multitude of viral proteins that modulate the immune response and inhibit various functions of model DCs including maturation and production of cytokines and chemokines. One such viral protein is γ134.5. This protein inhibits TLR mediated murine DC maturation by interacting with IKKɑ and IKKβ which leads to NF-κB inactivation, leading also to the inhibition of IL-6, IL-12, IFN-ɑ and IFN-β expression in immature DCs (88, 89). The late viral protein, *vhs*, has also been shown to decrease IFN-ɑ and IFN-β production by murine bone marrow derived DCs, amongst other DC proteins, as cells infected with a *vhs* deficient virus show increased levels of IFN-ɑ and IFN-β, compared to those infected with a wild type of virus (90). DCs infected with the wild type virus required endogenous IFNs to activate them compared to the *vhs* deficient virus (90). These studies need to be repeated in authentic human epidermal LCs and DCs.

### Plasmacytoid DCs

Plasmacytoid DCs (pDCs), differing in lineage from myeloid DCs, are the major producer of Type I IFNs, IFN-ɑ more than IFN-β, in the immunological response. In steady states pDCs are only found in blood and lymph nodes (44). Infection with HSV leads to pDCs infiltrating into sites of infection, such as the vagina, where they are seen in the upper dermis/lamina propria, including close to the basement membrane (44). pDCs respond directly to contact with HSV by producing Type I IFNs *via* endosomal TLR7, although they are not infected. Furthermore, HSV1 stimulation results in IL-18 production which contributes to IFN-γ production by NK cells (45). pDCs have also been shown to produce other cytokines and chemokines such as TNF, IL-6, CXCL10 and CCL3 resulting in recruitment and activation of NK cells and T cells (46).

### Macrophages

Macrophages are particularly important for the immune response against HSV contributing to a reduction in viral load in mouse models (91). Macrophages are recruited early into murine primary and human recurrent lesions by chemokines produced by keratinocytes (**Figure 2**) (6, 91). Resident macrophages are present in skin but their role in human HSV infection has not been sufficiently examined. The main function of macrophages during infection is phagocytosis of cell debris and secretion of cytokines including TNF, IFN-α, IL-6 and IL-12 (47, 92). IFN-α and -γ activate macrophages and modulate their cytokine production (93). Recent studies in the eyes of mice suggest that the major role of macrophages during HSV infection is to contribute to processing of HSV antigens and presentation to T cells, rather than directly degrading viruses, consistent with their deeper location than DCs (94). This is further supported by finding DC-SIGN^+^ mononuclear phagocytic cells clustering around infected apoptotic LCs in the dermis of human lesions (9, 95). Additionally, the M1/M2 phenotype of macrophages, determined by the cytokine environment, may impact on their role (96). In murine models, using different techniques in the same laboratory, the roles of macrophages appeared to differ (94, 97). In the most recent publication, comparing mice deficient in versus those overexpressing M2 macrophages, overexpression was associated with increased primary HSV replication in mouse eyes, increased latency and increased cytokine production. Yet in mice lacking M2 macrophages there was no difference in these parameters to wild type and no effect on eye disease. These studies seem to suggest the balance of M1/M2 macrophages may affect HSV induced cytokine production and eye disease but needs reproduction in human *ex vivo* systems.

### Natural killer cells

NK cells contribute to HSV clearance by two main mechanisms; direct cytotoxic effect on infected cells which have downregulated MHC-I expression, principally by the CD56^dim^ CD16+ subset and by releasing IFN-γ from the CD56^bright^ subset (98–100). As discussed above this IFN-γ is a major antiviral effector, enhancing MHC-I and -II expression on infected keratinocytes, activating macrophages and DCs and stimulating B cells. NK cells and γδ T cells are proposed to be the major producers of IFN-γ prior to T cell activation (48). IFN-γ induction in NK cells is also induced by IL-12 secretion by surrounding activated myeloid cells and continues in a powerful feedback loop prior to dampening of the immune response by regulatory T cells (49). The chemokine receptor expression of NK cells varies according to subtype (101, 102). CXCR1 and CXCR2 (receptors of IL-8) are expressed on CD56^dim^ NK cells while CXCR3, CCR5 and CCR7 are expressed on CD56^bright^ NK cells (103). This has important implications for which NK cell subsets home to particular sites during infection and the kinetics of doing so depending on the cascade of cytokines and chemokines produced over time. In addition to NK cells, decreased IFN-γ, IL-6 and IL-12 were observed in symptomatic mice where invariant natural killer T (iNKT) cells were lacking (104). The relative importance of iNKT cells in HSV infection is still poorly understood, although a role for them has been shown in mouse models (104). Therefore the relative roles of NK and iNKT cells in HSV infection should be further explored, partly in human *ex vivo* model systems.

### T cells

CD4 and CD8 T cells are a key part of the adaptive immune response against HSV. CD4 T cells are involved as early as 12-48 hours post infection in the immune response to recurrent human HSV infection and are also the most prominent effector cell by 4 days post onset of primary human lesions (6). CD4 T cells help clear HSV by helping DCs to activate CD8 T cells, shaping the immune response, and producing IFN-γ which limits HSV replication and spread and by activating B cells (50–52). The production of IFN-γ by CD4 T cells is crucial and is produced by Th1 CD4 T cells (105). Chemokines, such as CCL5, recruit CD4 T cells to sites of infection, where cytokines, such as IL-12 produced by DCs initiate the differentiation of CD4 T cells towards a Th1 subtype, producing IL-2 and IFN-γ (106, 107). This is crucial as IFN-γ production by CD4 T cells stimulates keratinocytes to secrete CXCL9 and CXCL10, which then recruit CD8 T cells to sites of infection (53, 54). These CD4 T cells can persist for >6 months after infection and are still capable of producing IFN-γ in response to HSV reactivation and release from intraepidermal sensory axons (108, 109). In mouse models of primary HSV1 infection, IFN-γ production has been shown to be essential in preventing vaginal infection (110), and in establishing and maintaining memory responses to HSV2 reinfection (111). These memory responses are driven by HSV specific CD4 Tissue Resident Memory cells (T_RM_s), as circulating CD4 memory T cells were unable to suppress viral replication (42). The key driver of this retention seems to be CCL5, which is highly upregulated in vaginal tissue following immunisation and infection (42).

In herpes keratitis, CD4 T cells and the cytokines they release are involved in both protection and ocular damage. CD4 T cells may infiltrate the cornea and conjunctiva and can become resident cells (112). As seen in vaginal infection CD4 Th1 cells secrete IFN-γ, as well as IL-2 in initial stages of ocular infection (55). These cytokines regulate neutrophil invasion into the cornea and prohibit CD4 Th17 cells, thus having a protective role (56). CD4 Th2 cells are also involved and secrete IL-10 and IL-4 which is crucial for corneal repair (55). In later stages of infection Th17 cells are observed and secrete IL-17 (56). These cells are involved in primarily stimulating other proinflammatory cytokines and not in limiting viral replication (113). As such these cells lead to the invasion of neutrophils into the cornea, which results in damage to the cornea (56). Mice lacking the IL-17 receptor showed a decreased severity of lesions as well as a decrease in CD4 T cells compared to WT mice (113, 114).

CD8 T cells infiltrate HSV lesions later than CD4 T cells, but their presence is associated with HSV clearance, indicating the vital role they play in the immune response against HSV (6, 7). CD8 T cells eliminate virally infected cells by either inducing apoptosis through Fas-FasL signalling or *via* perforin and granzyme B release (115). Importantly, CD8 T cells are also able to secrete antiviral IFN-γ in response to HSV infection. This also helps further promote DCs in presenting antigen to CD8 T cells and enhance MHC-I expression on HSV infected target cells (57). After HSV clearance CD8 T_RM_s remain at the dermo-epidermal junction as sentinels ready for HSV reactivation (116, 117). These cells are primed for encounters and express antiviral, chemotactic, and recruitment genes, and have the ability to produce perforin and granzyme B (118, 119). Studies of HSV lesions in the human female genital tract have shown that there is spatial heterogeneity of the CD8 T_RM_s and that these cells rely on IFN-γ for their antiviral effects rather than cytotoxicity. This means that HSV can utilise the gaps between the “spheres of IFN-γ” surrounding each of these cells to establish an infection (120, 121). Vaccine studies looking at eliciting CD8 T_RM_s in tissue have also suggested that IFN-γ is required for vaccine induced protection against HSV2 (122).

Regulatory T cells (Tregs) are a specialised cell of the adaptive immune response, involved in downregulating the immune response. Tregs use several mechanisms to modulate the immune response, such as the release of the immunomodulatory cytokines IL-10, TGF-β and IL-35 (123, 124). Studies of herpes keratitis and herpetic stromal keratitis have shown that Tregs play a key role in minimising damage to the cornea by preventing generation, migration, or effects of pathogenetic T cells to the cornea, shown by worsening conditions of keratitis upon depletion of these cells (125). Indeed at least part of this mechanism Tregs occurs *via* production of IL-10, which suppresses the proliferation of CD4 and CD8 T cells, and the production of inflammatory cytokines and chemokines (e.g., IL-2, IL-6, CCL3) (58, 59).

### B cells

In human vaccine studies systemic anti-HSV antibody has been implicated in protection against genital herpes (126) and in murine eye infection models a role for systemic antibody diffusion into the cornea, albeit slow to equilibrate has also been shown (127). The role of locally infiltrating B cells in HSV infection and other sexually transmitted viruses is less clear than that of other infiltrating adaptive immune cells. However recent studies show B cells immigrate to sites of infection by HSV, stimulated by CCL19, CCL21 and CXCL12 and CXCL13 through CCR7 and CXCR4 receptors, respectively. Antibody secreting cells and detectable and increasing levels of neutralising anti-HSV antibodies were present in lesion biopsies and clustered with CD4 T cells suggesting crosstalk (128–131). However circulating high levels of binding and neutralising antibodies within human blood are not proportional to the frequency or severity of recurrent lesions, and vaccine induced antibodies to gD were not protective against genital HSV2 in seronegative subjects (132). Several animal models indicate that local B cell recruitment is necessary for efficient HSV clearance (52, 133, 134). For comparison HIV infected human foreskin also contained antibody secreting B cells close to the dermo-epidermal junction (135), further supporting a local effect of B cell antibody secretion which may contribute to viral clearance in the genital mucosa in humans. In another site, in mouse models of recurrent ocular HSV there were increased frequencies of systemic memory B cells and of antibody secreting plasma cells in the trigeminal ganglion in mice with asymptomatic compared to symptomatic eye infection (136).

Beyond their antibody response, B cells have been observed to secrete IFN-γ, IL-4, IL-6, and a host of other cytokines (137, 138). However, within the context of HSV infection the contribution of these cytokines by activated B cells in lesions is likely to be negligible compared to other immune cells in lesions, considering the limited number of B cells present (6).

## The chemokine and cytokine response in mucosal tissue

**Figure 5:**
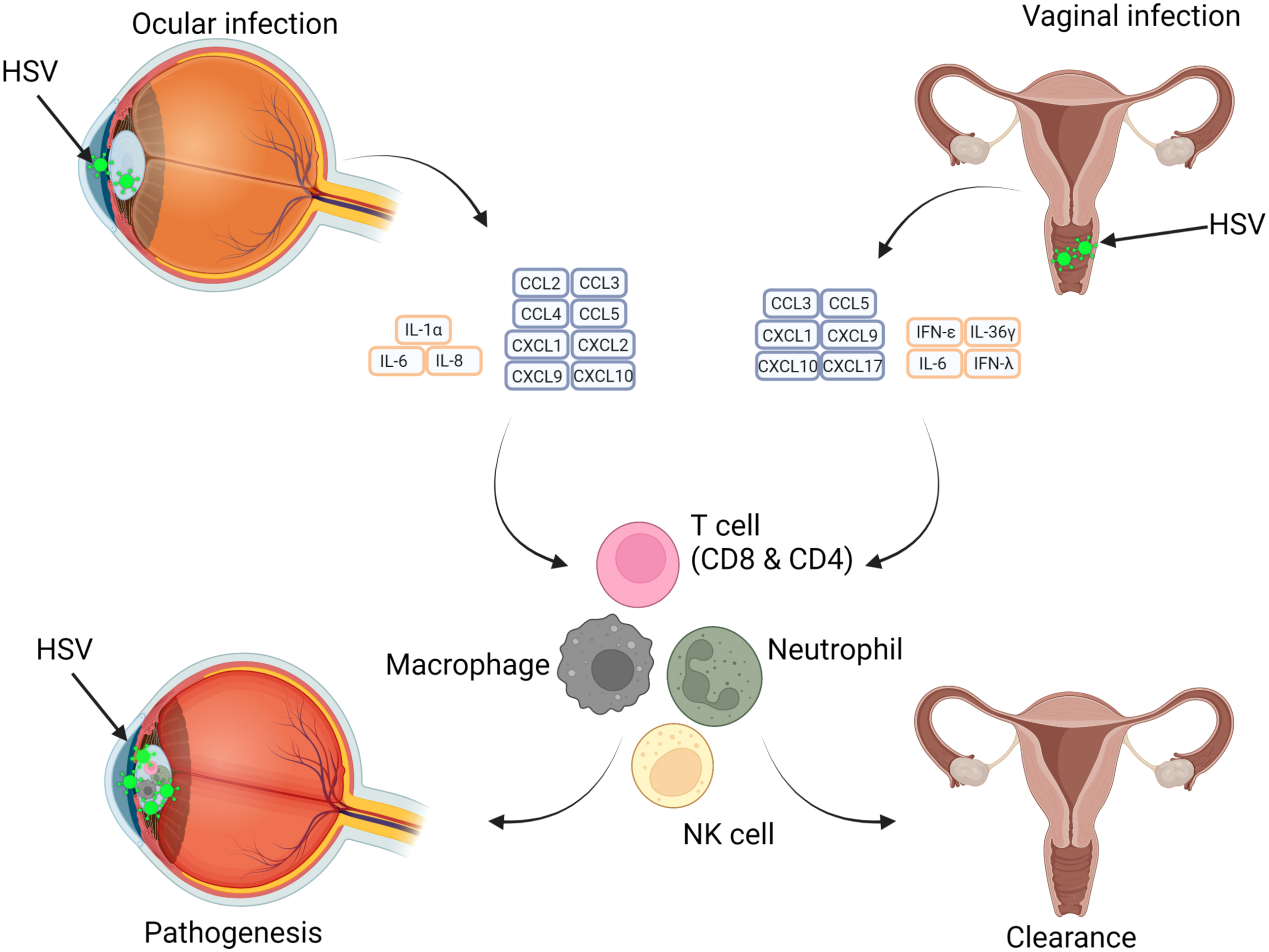
Differences in HSV infection of the Eye and Vagina in relation to immunopathology and protective abilities of cytokines and chemokines. HSV infection pathogenesis is site dependent with tissue-specific immune cell responses influencing this. Ocular infection results in a cytokine storm which results in adaptive and innate immune cell recruitment and further damage. In vaginal infection, similar subsets of immune cells are recruited, however the impact is beneficial and results in resolution of infection. The varying effects of cytokines impacts the outcome at different anatomical regions.

The sections above considered the role of individual cell types in producing and responding to cytokines and chemokines in response to HSV infection, especially in lesions. How are these integrated into tissue-wide responses? In this section we will examine and compare specific immune responses to HSV in the eye, usually infected with HSV1, and the vagina, infected by either HSV2 or HSV1, and the roles of cytokines and chemokines. **Figure 5** summarises the differences between the responses to cytokines and chemokines in the eye and vagina, while **Table 2** presents a summary of the cytokines and chemokines involved in ocular and vaginal HSV infection.

**Table 2:** Summary of known cytokines and chemokines involved in HSV infections of Eye and Vagina and their functions.

Cytokines/Chemokines	Effect	Function	References
**Eye**			
*Cytokines*			
IL-6	Immunopathologic	Increases CCL3 and CCL5 expression which leads to neutrophil and NK cell infiltration and inflammation	(139)
IL-1α	Immunopathologic	Promotes leukocyte infiltration into the cornea	(42, 140)
*Chemokines*	**Effect**	**Function**	**References**
CCL3	Immunopathologic	Increases infiltration of CD4 T and neutrophils into the cornea and may affect CXCL2 expression	(6)
CCL5	Immunopathologic	Recruitment of NK cells, neutrophils, and monocytes into the cornea	(42)
CXCL10	Protective	Attracts CD8 T cells into the cornea	(42, 141)
CXCL9	Immunopathologic	Accelerates Herpes Keratitis as it increases neutrophils and CD4 T cells in the cornea leading to an increased inflammatory response	(142, 143)
CXCL1	Unknown	Produced during recurrent infection and attract neutrophils to the cornea	(144, 145)
**Vagina**			
*Cytokines*	**Effect**	**Function**	**References**
IFN-Ɛ	Protective	Constitutively expressed by the female reproductive tract. Mice lacking IFN-Ɛ showed an increased replication of HSV2	(146, 147)
IL-36γ	Protective	Limits HSV replication and induces CCL20 and CXCL1 leading to neutrophil infiltration	(69, 148)
*Chemokines*	**Effect**	**Function**	**References**
CXCL1	Protective	Targets neutrophils causing migration and activation of these cells	(149)
CXCL17	Protective	Contributes to the migration of CD44^high^CD62^low^CD8 T_EM_s and establishment of CD103^high^CD8 T_RM_s in vaginal tissue infected with HSV1	(150)
CCL3	Protective	Drives the production of IFN-γ	(151)
CCL5	Protective	Influences NK cell migration into the vagina	(152)
CXCL9	Protective	Involved in mobilisation of NK and cytotoxic T cells	(153)
CXCL10	Protective	Involved in mobilisation of NK and cytotoxic T cells	(153)

### Local cytokine and chemokine responses seen in the eye in response to HSV infection

HSV1 infection of the eye is a serious disease, with herpes keratitis as one of the leading causes of infectious blindness in the world (154). Therefore it is crucial to understand how HSV interacts with cells in the eye and the role of cytokines and chemokines in influencing the pathology of herpes keratitis. HSV1 infection of the murine corneal epithelium leads to the production of many cytokines and chemokines, such as IL-8, CCL2, CCL3, CCL4, CCL5, CXCL1, CXCL2 and CXCL10 (141, 155, 156). These signals are key for recruitment of leukocytes into the cornea (142). RNA expression for CCL3, CCL4, CCL5 and CCL2 have been shown to persist into the latent stage of ocular HSV1 infection, i.e., longer than 30 days post infection (143). This prolonged activation of immune cells can cause extensive damage to the eye, resulting in the immunopathology of the disease. This is also reflected in the fact that the RNA expression of some of the corresponding chemokine receptors are still highly expressed in infected corneas, past 30 days post infection (143). Studies have shown that some of these chemokines and their receptors are key drivers of the immunopathology as their reduction or absence leads to a reduction of symptoms. Mice deficient in CCL3 showed a decrease in blindness, reduction in infiltrating CD4 T cells and neutrophils and lower levels of CXCL2 expression when infected with herpes keratitis (157). Other examples of these are CXCR3 and CCR5, where deficiency of these receptors also leads to less severe disease (144).

Most chemokines produced in herpes keratitis are detrimental except it seems for CXCL10. CXCL10 is a homeostatic and protective chemokine that is expressed by epithelial cells (55). CXCL10 controls the inflammatory response through attracting CD8 T cells, NK cells, monocytes and Th1 CD4 T cells (55). Indeed, mice lacking CXCL10 and CXCR3 (the receptor for CXCL10) showed a decrease in CD8 T cells and increased severity of herpes keratitis, compared to WT mice (145, 158). HSV specific CD8 T cell numbers could be restored in the cornea by application of CXCL10 (145). CXCL9, another ligand for CXCR3 produced by corneal cells in the absence of CXCL10, seems to show the opposite function to CXCL10, as it accelerates herpes keratitis progression (139, 140). This immunopathologic effect may be because CXCL9 can aggregate neutrophils to cause an inflammatory response (140) and increase the number of CD4 T cells infiltrating into the cornea. This is supported by experiments showing mice deficient in CXCL9 had a decrease of CD4 T cell infiltration in the cornea (159). These observations highlight the contrasting function of two chemokines which both target CXCR3, with extremely different pathological outcomes. CXCL1, the only chemokine produced by the central corneal epithelium, attracts neutrophils to the cornea but is not involved in clearing HSV (149, 160). CXCL1 is only produced by corneal stromal cells in recurrent herpes keratitis rather than primary infection (150).

Several cytokines are also involved in herpes keratitis immunopathology, such as IL-6 and IL-1α. IL-6 has been seen in mouse HSV infected corneas, and is induced by IL-17 (70). IL-6 has been observed to increase CCL3 and CCL5 expression which supports neutrophil and NK cell infiltration and increased corneal inflammation, without reducing viral load (151). The key role of IL-6 in herpes keratitis immunopathology was demonstrated in mice lacking IL-6; they had decreased corneal opacity, inflammation, and neutrophil recruitment (151). IL-6 regulates the balance of Tregs and Th17 cells, as IL-6 production leads to Th17 differentiation and Treg inhibition, which could lead to more severe HSV infection and ocular damage (154). IL-1α is another cytokine that is produced by HSV infected corneal epithelium (55). IL-1α works together with IL-6 to promote leukocyte infiltration into the cornea (153).

### Local cytokine and chemokine responses seen in the vagina in response to HSV infection

Genital herpes is mainly caused by HSV2 infection of genital mucosal tissues, such as the vagina. However HSV1 is now also a major cause of (initial) genital herpes (152) (126). Chemokines and cytokines play a vital role in the immune response of these tissues, such as mobilising NK cells and T cells to sites of infection where they limit viral spread and disease. Minimal work has been conducted on other genital tissues and human tissues, so this section will mostly focus on HSV infection in the murine vagina.

Many chemokines are produced in the murine vaginal mucosa, some specifically by epithelial cells in the mucosal tissue. CXCL1 is one such chemokine that is produced by epithelial cells of the female genital tract in response to HSV2 infection (161). CXCL1 targets neutrophils, through the CXCR2 receptor, and leads to the migration and activation of these cells (146). Another chemokine constantly produced in the vagina is CXCL17 (147). CXCL17 contributes to the migration of CXCR8 expressing CD44^high^CD62^low^CD8 T Effector Memory cells (T_EM_s) and establishment of CD103^high^CD8 T_RM_s in vaginal tissue infected with HSV1 (147). Other chemokines are also expressed in HSV infected vaginas, such as CCL3 and CCL5 (69, 162). These chemokines are important for recruitment of other immune cells, such as NK cells. CCL5 specifically seems to drive NK cell recruitment during HSV2 vaginal infection, as loss of CCL5 leads to a decrease in infiltrating NK cells and increased viral loads, indicating the importance of NK cells in HSV2 immunity (148). Other important chemokines are CXCL9 and CXCL10. Both chemokines are involved in the mobilisation of NK and cytotoxic T cells (69). Mice deficient in either CXCL9 or CXCL10 had increased viral titres, reduction in immigrating cells (i.e., NKs) increased inflammation and mortality (69). Mice deficient in CXCR3 (the receptor for CXCL9 and CXCL10) are also sensitive to HSV2 infection with increases in viral titres and mortality. Interestingly CD8 T cells are still able to migrate to sites of infection in these mice but there were defects in their effector functions (163).

Cytokines are also important in controlling HSV vaginal infection, especially cytokines produced by epithelial cells. IFN-Ɛ is a Type I IFN that is constitutively expressed by the female reproductive tract (164, 165). Mice lacking IFN-Ɛ showed an increase in HSV2 replication compared to WT mice, indicating that IFN-Ɛ plays a role in HSV2 immunity (165). IFN-λ (Type III IFN) has also been shown to restrict HSV replication in mucosal epithelial cells (166). IL-36γ is an inflammatory mediator at epithelial sites (167). It has also been shown to be expressed in a human vaginal epithelial cell model that was infected with HSV2 (167). In mouse models, pre-treatment with IL-36γ also led to decreased HSV2 replication, limited viral replication, delayed disease onset, decreased severity, and increased survival (167). Pre-treatment also led to the production of proinflammatory cytokines and chemokines including CCL20 and CXCL1 (167) which induce neutrophil infiltration (168). IL-36γ deficient mice develop genital diseases more rapidly, have decreased survival, reduced neutrophil recruitment and CXCL1 and CCL20 expression (168).

Apart from tissue specific cytokines and chemokines, there are many similarities between the cytokines and chemokines produced by the vagina and the eye. However, these similarities are only in terms of production and not in role. For example, CCL3, CCL5 & CXCL9 are found in both eye and vaginal HSV infections, but in the eye these chemokines are known to cause the immunopathology of herpes keratitis, while in the vagina these chemokines have a protective role. Some cytokines are also exclusively produced by the vagina (IFN- Ɛ and IL-36γ), and thus would only play a role in HSV infection in female genital tracts. This highlights that there are differences in how the immune response is shaped against HSV at different sites of infection, and how a ‘one size fits all’ approach to immunotherapy may not work for all areas where HSV infects. This will also need to be considered when developing new vaccines for HSV. Is it also important to note that most of these studies have been conducted in mice, which while an important resource, may not match the cytokines and chemokines produced by humans.

## What role chemokines and cytokines play in the development of HSV vaccines

Currently there is still no licensed human prophylactic or therapeutic vaccine for HSV as even the most promising vaccine candidates have only shown partial efficacy in clinical trials. We previously wrote a comprehensive review of HSV vaccine candidates and their current development status and the outcomes of clinical trials (169). All prophylactic vaccines that have so far been trialled have only been able to stimulate neutralising antibodies and CD4 T cell induction. However CD8 T cells are crucial for clearance of HSV from lesions (7).

Incorporation of chemokines and cytokines into DNA vaccines or topical application after vaccination may help attract and stimulate CD4 and CD8 T cells. Indeed, HSV vaccination using the addition of chemokines and cytokines to a DNA vaccine, has been shown to be effective in various murine models. The addition of CCL19 or CCL28 to a HSV2 gD DNA vaccine has been shown to induce long lasting HSV2 specific immunity, such as IgA, IgG and IgM responses, cytokine induction and CCR3+ T cell enrichment in spleens (170), protecting against a lethal HSV2 vaginal challenge in mice (171). Another study also utilised IL-28 in a HSV2 DNA vaccine, leading to increased Th1 and Th2 cytokines, increased neutralising antibodies, decreased viral loads and IFN-γ production from splenocytes, compared to a HSV2 gD DNA vaccine without IL-28 (172). Other studies have utilised cytokine/chemokines adjuvants such as IL-12, IL-21 and CCL3 to increase efficacy. These vaccines have been shown to protect mice from vaginal challenge from HSV2 and were able to induce a strong T cell response to clear the virus in the major site of infection, vagina, and in draining lymph organs (170).

Another vaccine strategy involves using attenuated HSV vaccines and topical chemokine application, in a ‘prime and pull’ method, to recruit and establish a pool of T cells within the peripheral tissue. This approach requires conventional vaccination to elicit systemic T cell responses (prime) followed by the application of topical chemokines to recruit activated T cells to the genital tract and thus establish a pool of long term and protective T cells (pull). In this study mice were vaccinated with an attenuated HSV2 vaccine and then treated with topical vaginal application of CXCL9 and CXCL10. This method led to elevated levels of CD4 and CD8 T cells seen in the vaginal mucosa, with CD8 T cells persisting for over 12 weeks. When presented with a lethal HSV2 challenge, mice had a 100% survival rate, and did not show clinical symptoms (173). Another strategy is to construct a recombinant HSV vaccine where chemokines are expressed by the virus. One study utilised IL-12p35 or IL-12p40, inserted into HSV1 KOS virus as a vaccine for herpes keratitis. Mice immunised with the IL-12p35 insert virus showed decreased viral titres, rapid viral clearance, high neutralising antibody levels, high cytotoxic T cell levels and IFN-γ levels (174). However little quantitative analysis was conducted to determine the efficacy of this vaccine in terms of viral titres in the cornea or ocular health. Therefore the efficacy needs to be more rigorously studied, compared to other vaccines in clinical trials and in other animal models. Nevertheless, these studies indicate that the addition of cytokines or chemokines to vaccines can result in successful protection in mouse models. Such strategies now need to be further developed for and selected for trialling in humans.

## Concluding remarks

In conclusion, cytokines and chemokines are an integral part of the immune response against HSV, as they drive the recruitment and effector functions of many immune cells. However, these responses have been mostly overlooked in vaccine and drug design in the past. Only now is work being conducted on vaccines that specifically utilise cytokines and chemokines to stimulate cells needed for a strong response against HSV. Work on cytokines and chemokines has also sometimes focused on the ligands secreted in tissue infections, but not defined which cells are secreting these factors. This is essential to unravel the key immune cell interactions needed for optimal protection against HSV infection and therefore, to guide development of vaccines and immunotherapies. Since obtaining human samples from key sites of HSV infection such as the vagina and eyes can be difficult, most work has been conducted in murine models or blood derived human cells. Where possible, research must be conducted in human anogenital and corneal tissue samples to ensure the responses seen in mice *in vivo* and human cells *in vitro* truly represents the human *in vivo* setting, particularly as there are anatomical differences in the induced chemokines and cytokines, as reviewed here for the vagina and the eye. However, we acknowledge the fact that murine models and blood derived human cells provide a reasonable guide for work in human anogenital and corneal tissues as these samples are rare and limiting.

## Author contributions

JS and JH are equal first authors. AC is the corresponding author. JS, JH, NT, and AC all contributed to editing the manuscript. All authors contributed to the article and approved the submitted version.

## Funding

Our research was funded by the Australian National Health and Medical Research Council (NHMRC) (APP 1163748 & APP1177942).

## Acknowledgments

All figures were created with Biorender.com.

## Conflict of interest 

AC reports other funding to his institution from GSK, Merck and BioCSL/Sequiris, outside the submitted work.

The remaining authors declare that the research was conducted in the absence of any commercial or financial relationships that could be construed as a potential conflict of interest.

## Publisher’s note

All claims expressed in this article are solely those of the authors and do not necessarily represent those of their affiliated organizations, or those of the publisher, the editors and the reviewers. Any product that may be evaluated in this article, or claim that may be made by its manufacturer, is not guaranteed or endorsed by the publisher.

## Abbreviations

HSV, Herpes Simplex Virus; HIV, Human Immunodeficiency Virus; IFN, Interferon; LC, Langerhans Cell; DC, Dendritic Cell; TNF, Tumour Necrosis Factor; GM-CSF, Granulocyte Macrophage-Colony Stimulating Factor; PRR, Pattern Recognition Receptor; TLR, Toll-Like Receptor; cDC, Conventional Dendritic Cell; MDDC, Monocyte Derived Dendritic Cell; pDC, Plasmacytoid Dendritic Cell; NK, Natural Killer Cell; T_RM_, Tissue Resident Memory T Cell; Treg, T Regulatory Cell; T_EM_, T Effector Memory Cell.
